# Evolution of Ubiquinone Biosynthesis: Multiple Proteobacterial Enzymes with Various Regioselectivities To Catalyze Three Contiguous Aromatic Hydroxylation Reactions

**DOI:** 10.1128/mSystems.00091-16

**Published:** 2016-08-30

**Authors:** Ludovic Pelosi, Anne-Lise Ducluzeau, Laurent Loiseau, Frédéric Barras, Dominique Schneider, Ivan Junier, Fabien Pierrel

**Affiliations:** a Laboratoire Technologies de l’Ingénierie Médicale et de la Complexité—Informatique, Mathématiques et Applications, Grenoble (TIMC-IMAG), University of Grenoble Alpes, Grenoble, France; b Centre National de Recherche Scientifique (CNRS), TIMC-IMAG, UMR5525, Grenoble, France; c School of Fisheries and Ocean Sciences, University of Alaska—Fairbanks, Fairbanks, Alaska, USA; d Aix-Marseille Université, CNRS, Laboratoire de Chimie Bactérienne, UMR 7283, Institut de Microbiologie de la Méditerranée, Marseille, France; University of California San Diego

**Keywords:** biosynthesis, evolution, proteobacteria

## Abstract

The ubiquitous ATP synthase uses an electrochemical gradient to synthesize cellular energy in the form of ATP. The production of this electrochemical gradient relies on liposoluble proton carriers like ubiquinone (UQ), which is used in the respiratory chains of eukaryotes and proteobacteria. The biosynthesis of UQ requires three hydroxylation reactions on contiguous positions of an aromatic ring. In *Escherichia coli*, each of three UQ flavin monooxygenases (FMOs), called UbiF, UbiH, and UbiI, modifies a single position of the aromatic ring. This pattern of three hydroxylation reactions/three proteins has been accepted as a paradigm in UQ biology. Using a phylogenetic analysis, we found that UbiF, UbiH, and UbiI are detected only in a small fraction of proteobacteria, and we identified two new types of UQ FMOs: UbiM, which is distributed in members of the alpha, beta, and gamma classes of proteobacteria, and UbiL, which is restricted to members of the alphaproteobacteria. Remarkably, the *ubiL* and *ubiM* genes were found in genomes with fewer than three UQ hydroxylase-encoding genes. We demonstrated, using biochemical approaches, that UbiL from *Rhodospirillum rubrum* and UbiM from *Neisseria meningitidis* hydroxylate, respectively, two and three positions of the aromatic ring during UQ biosynthesis. We conclude that bacteria have evolved a large repertoire of hydroxylase combinations for UQ biosynthesis, including pathways with either three specialist enzymes or pathways with one or two generalist enzymes of broader regioselectivity. The emergence of the latter is potentially related to genome reduction events.

**IMPORTANCE** UQ, a key molecule for cellular bioenergetics that is conserved from proteobacteria to humans, appeared in an ancestral proteobacterium more than 2 billion years ago. UQ biosynthesis has been studied only in a few model organisms, and thus, the diversity of UQ biosynthesis pathways is largely unknown. In the work reported here, we conducted a phylogenomic analysis of hydroxylases involved in UQ biosynthesis. Our results support the existence of at least two UQ hydroxylases in the proteobacterial ancestor, and yet, we show that their number varies from one to four in extant proteobacterial species. Our biochemical experiments demonstrated that bacteria containing only one or two UQ hydroxylases have developed generalist enzymes that are able to catalyze several steps of UQ biosynthesis. Our study documents a rare case where evolution favored the broadening of an enzyme’s regioselectivity, which resulted in gene loss in several proteobacterial species with small genomes.

## Abstract (precis)

UQ, a key molecule for cellular bioenergetics that is conserved from proteobacteria to humans, appeared in an ancestral proteobacterium more than 2 billion years ago. UQ biosynthesis has been studied only in a few model organisms, and thus, the diversity of UQ biosynthesis pathways is largely unknown. In the work reported here, we conducted a phylogenomic analysis of hydroxylases involved in UQ biosynthesis. Our results support the existence of at least two UQ hydroxylases in the proteobacterial ancestor, and yet, we show that their number varies from one to four in extant proteobacterial species. Our biochemical experiments demonstrated that bacteria containing only one or two UQ hydroxylases have developed generalist enzymes that are able to catalyze several steps of UQ biosynthesis. Our study documents a rare case where evolution favored the broadening of an enzyme’s regioselectivity, which resulted in gene loss in several proteobacterial species with small genomes.

## INTRODUCTION

Isoprenoid quinones are encountered in almost all living organisms, where they function mainly as electron and proton transporters in photosynthetic and respiratory chains (1). The content of isoprenoid quinones varies between bacterial species, and isoprenoid quinone profiles have therefore been used as taxonomic tools (2, 3). Isoprenoid quinones contain a polar redox-active head group coupled to a lipid side chain that varies in both length and degree of saturation. The main isoprenoid quinones are menaquinone (MK) and ubiquinone (UQ), which are distinguished by the structure of the head group, naphthalene ring and benzene ring, respectively. Eukaryotes synthesize UQ, and humans and rodents additionally possess MK_4_, which is also known as vitamin K (1). In microorganisms, MK is the most widespread quinone, since it is present in members of both *Bacteria* and *Archaea*, whereas UQ is restricted to members of the alpha-, beta-, and gammaproteobacteria (1, 2, 4).

Schoepp-Cothenet et al. suggested that the innovation of UQ occurred in the common ancestor of the alpha-, beta-, and gammaproteobacteria (5) after the rise of oxygen in the terrestrial atmosphere during the Great Oxidation Event, 2.4 billion years ago (6). At that time, living organisms faced a tremendous bioenergetic challenge owing to the propensity of MK to become nonenzymatically oxidized at a high rate when exposed to oxygen. This biochemical property likely impaired the electron carrier function of MK in respiratory chains, which might have been compensated for by the innovation of UQ, which has a higher positive redox midpoint potential and, thus, greater resistance to oxidation (5). Interestingly, a few genes of the UQ biosynthesis pathway (*ubiA*, *ubiX*, and *ubiD*) have recently been suggested to derive from the futalosine pathway (7), which is one of the two routes used by microorganisms to synthesize MK (4, 8). Therefore, MK existed before UQ, and today, MK and UQ still function predominantly in anaerobic and aerobic respiratory chains, respectively (1).

**FIG 1:**
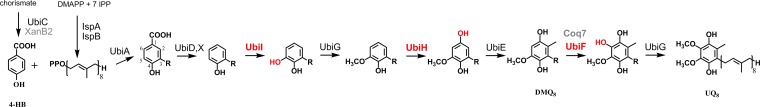
Biosynthetic pathway of ubiquinone 8 (UQ_8_) in *E. coli*. The numbering of the aromatic carbon atoms used throughout this study is shown for the product of the prenylation reaction catalyzed by UbiA. The octaprenyl tail is represented by R on carbon 3 of the different biosynthetic intermediates. Enzymes catalyzing the hydroxylation reactions are shown in red (UbiI, UbiH, and UbiF), and those replacing Ubi proteins in some proteobacteria are in grey (XanB2 and Coq7). DMAPP, dimethylallyl pyrophosphate; IPP, isopentenyl pyrophosphate; 4-HB, 4-hydroxybenzoic acid; DMQ_8_, C-6-demethoxy-ubiquinone 8; UQ_8_, ubiquinone 8.

The biosynthetic pathway of UQ has been elucidated mostly by studying the gammaproteobacterium *Escherichia coli* and the eukaryote *Saccharomyces cerevisiae*, which synthesize, respectively, UQ_8_ and UQ_6_ (with side chains of eight and six isoprene units) (9, 10). Overall, UQ biosynthesis requires one prenylation, one decarboxylation, three hydroxylation, and three methylation reactions to transform the precursor of the benzoquinone head group, 4-hydroxybenzoic acid (4-HB), into UQ (Fig. 1). In *E. coli*, the three hydroxylation reactions are catalyzed on the carbon atoms C-1, C-5, and C-6 of the aromatic ring by three different enzymes, called monooxygenases or hydroxylases, namely, UbiH, UbiI, and UbiF (Fig. 1) (1, 11). All three proteins share 29 to 38% sequence identity and belong to the class A flavoprotein monooxygenases (FMOs). Class A FMOs are a widely distributed subset of FMOs that use a flavin adenine dinucleotide (FAD) cofactor, NAD(P)H as the electron donor, and dioxygen in order to catalyze aromatic hydroxylation reactions (12). Class A FMOs are characterized by a specific DG amino acid sequence motif with a dual function in both FAD and NAD(P)H binding (12).

In eukaryotes, only two hydroxylases, Coq6 and Coq7, have been shown to participate in UQ biosynthesis so far (13–16). Coq6 belongs to the class A FMOs and hydroxylates C-5 (14, 17), while Coq7 is a di-iron monooxygenase which hydroxylates C-6 (16, 18). The C-1 hydroxylase has yet to be characterized. Interestingly, the two gammaproteobacteria *Pseudomonas aeruginosa* and *Thiobacillus ferrooxidans* (also called *Acidithiobacillus ferrooxidans*) lack *ubiF* but possess instead a Coq7 homolog, which has been shown to complement the C-6 hydroxylation defect of an *E. coli* strain in which *ubiF* is deleted (16). Hence, the UbiF and Coq7 monooxygenases perform the same function despite using different cofactors and being unrelated. Our current view of UQ biosynthesis is therefore limited to a small number of species and postulates the requirement of three distinct hydroxylases, each catalyzing the hydroxylation of a single C position.

We scrutinized 67 representative bacterial genomes to both determine the distribution of the UbiF-, UbiH-, UbiI-, and Coq7-encoding genes within the phylum *Proteobacteria* and explore the diversity of the hydroxylation systems used for UQ biosynthesis. By combining phylogenomic inferences based on *in silico* homology searches and heterologous functional complementation assays in *E. coli*, we identified two new FMOs involved in UQ synthesis (UQ FMOs), called UbiL and UbiM. This raises to five the number of UQ FMOs and to six the number of distinct hydroxylases involved in this biosynthetic pathway. Surprisingly, we found that several proteobacterial genomes contained only one or two UQ hydroxylase-encoding genes. The *ubiL* or *ubiM* gene was almost always present in these genomes, and we indeed demonstrated that the corresponding UbiL and UbiM proteins were able to hydroxylate two and even three positions of the UQ head group. We thus revisited the current postulate of the requirement of three different monooxygenases to hydroxylate the three positions of the UQ head group, and we provide a likely scenario explaining the evolution of the five UQ FMOs. More generally, our study documents the evolution of specialist and generalist enzymes—able to hydroxylate one or several positions, respectively—within the same protein family.

## RESULTS

### Two new potential FMOs involved in UQ biosynthesis.

We analyzed the genomes of 67 representative species of alpha-, beta-, and gammaproteobacteria, which are the only three subclasses of UQ producers known so far in bacteria. We performed BLAST searches in the NCBI database using the sequences of UbiF, UbiH, and UbiI from *E. coli* and Coq7 from *P. aeruginosa* as references. Phylogenetically distant sequences still clustering with the hydroxylase clades were then used as further queries to explore the full sequence space of UQ hydroxylases (see Table S1 in the supplemental material).

10.1128/mSystems.00091-16.7 Table S1  Genome sizes of the proteobacteria used in this study and GenBank accession numbers of the protein sequences (NCBI, http://www.ncbi.nlm.nih.gov). Download Table S1, PDF file, 0.1 MB. Copyright © 2016 Pelosi et al. 2016 Pelosi et al. This content is distributed under the terms of the Creative Commons Attribution 4.0 International license.

**FIG 2:**
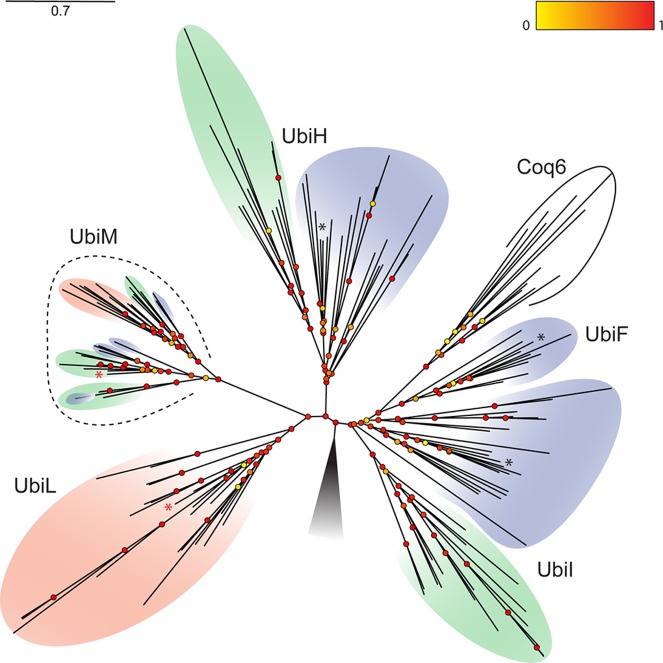
Molecular phylogeny of the FMOs involved in UQ biosynthesis. The phylogenetic tree was constructed using the maximum-likelihood method. Proteins found in alpha-, beta- and gammaproteobacteria are highlighted in pink, green, and blue, respectively. The names of the different hydroxylases are indicated next to the different clades. Black asterisks designate *E. coli* sequences, and red ones designate the FMO sequences of *R. rubrum* and *N. meningitidis* characterized in this study. The outgroup is symbolized by a shaded triangle. The results of the approximate likelihood ratio test for nodes are presented here by circles colored in a gradient from yellow to red to represent values from 0 to 1. The scale bar indicates the average number of substitutions per site.

Surprisingly, we detected one to four UQ FMOs per genome, and we found *coq7* genes in the genomes of species outside the gamma subclass of proteobacteria to which *A. ferrooxidans* and *P. aeruginosa* belong. The poor phylogenetic signal obtained with the short Coq7 primary sequences (~170 to 210 amino acids) prevented a deeper phylogenetic analysis, and therefore, we were unable to infer any hypothesis of the evolution of this protein. In contrast, we were able to construct a phylogenetic tree using the sequences of UQ FMOs, which contain about 400 amino acids (Fig. 2). To root the tree, more distant proteobacterial FMOs were included as an outgroup. We also had a goal of identifying the ancestor of Coq6, which is a UQ FMO unique to eukaryotes, so we included 11 Coq6 sequences (see Table S1 in the supplemental material). Based on UQ biosynthesis in *E. coli*, we expected to retrieve a global proteobacterial tree topology displaying four sequence clades, including one for each of the UbiF, UbiH, and UbiI proteins and the outgroup. However, the analyses conducted with both maximum-likelihood (Fig. 2) and Bayesian (see Fig. S1) algorithms revealed (i) the existence of five sequence clades, (ii) a monophylum of *ubiI*, *ubiF*, and *COQ6*, (iii) a lack of *ubiF*, *ubiH*, and *ubiI* within the alphaproteobacterial genomes, (iv) a rooting position located between the UbiF-UbiI-Coq6 clade and the other clades, and (v) the existence of two protein clades unrelated to the well-established *E. coli* UQ hydroxylases and hitherto unknown (Fig. 2). One of these two clades contains exclusively proteins from alphaproteobacteria, and we decided to call it UbiL, while the UbiM clade encompassed the three alpha-, beta- and gammaproteobacterial subclasses. The UbiM and UbiL protein sequences contained the FAD/NAD(P)H fingerprint motifs typical of FMOs (19) (see Fig. S2).

10.1128/mSystems.00091-16.1 Figure S1  Molecular phylogeny of FMOs involved in UQ biosynthesis. The phylogenetic tree was constructed using the Bayesian algorithm. Proteins found in alpha-, beta- and gammaproteobacteria are highlighted in pink, green, and blue, respectively. The names of the different hydroxylases are indicated next to the different clades. Black asterisks designate *E. coli* sequences. Red asterisks designate the FMO sequences of *R. rubrum* and *N. meningitidis* characterized in this study. The outgroup is symbolized by a shaded triangle. Posterior probabilities for nodes are presented here by circles colored in a gradient from yellow to red, representing values between 0% and 100%, respectively. The scale bar indicates the average number of substitutions per site. Download Figure S1, TIF file, 0.7 MB. Copyright © 2016 Pelosi et al. 2016 Pelosi et al. This content is distributed under the terms of the Creative Commons Attribution 4.0 International license.

10.1128/mSystems.00091-16.2 Figure S2  Sequence alignment of FMOs involved in UQ biosynthesis in *E. coli* K-12 (UbiI, NP_417382; UbiF, NP_415195; and UbiH, NP_417383), in *R. rubrum* (UbiL_Rr_, YP_428788), and in *N. meningitidis* (UbiM_Nm_, EFM05404). Alignments were generated using Clustal Omega and analyzed with Jalview version 2.0.1 (A. M. Waterhouse, J. B. Procter, D. M. Martin, M. Clamp, and G. J. Barton, Bioinformatics **25**:1189–1191, 2009, http://dx.doi.org/10.1093/bioinformatics/btp033). The red bars indicate the first FAD fingerprint sequence, which includes the well-known Rossmann fold (containing the GXGXXG motif), the DG amino acid sequence, which is proposed to play a dual function in both FAD and NAD(P)H binding, and the second FAD binding motif, containing the GD sequence with the highly conserved aspartyl residue that contacts the O-3′ of the ribose moiety of FAD (M. H. Eppink, H. A. Schreuder, and W. J. Van Berkel, Protein Sci **6**:2454–2458, 1997, http://dx.doi.org/10.1002/pro.5560061119). The height of the bar and the size of the letter below the alignment reflect the conservation of a particular amino acid and the consensus sequence, respectively. Download Figure S2, PDF file, 0.5 MB. Copyright © 2016 Pelosi et al. 2016 Pelosi et al. This content is distributed under the terms of the Creative Commons Attribution 4.0 International license.

### Diverse combinations of UQ hydroxylases exist in proteobacteria.

**FIG 3:**
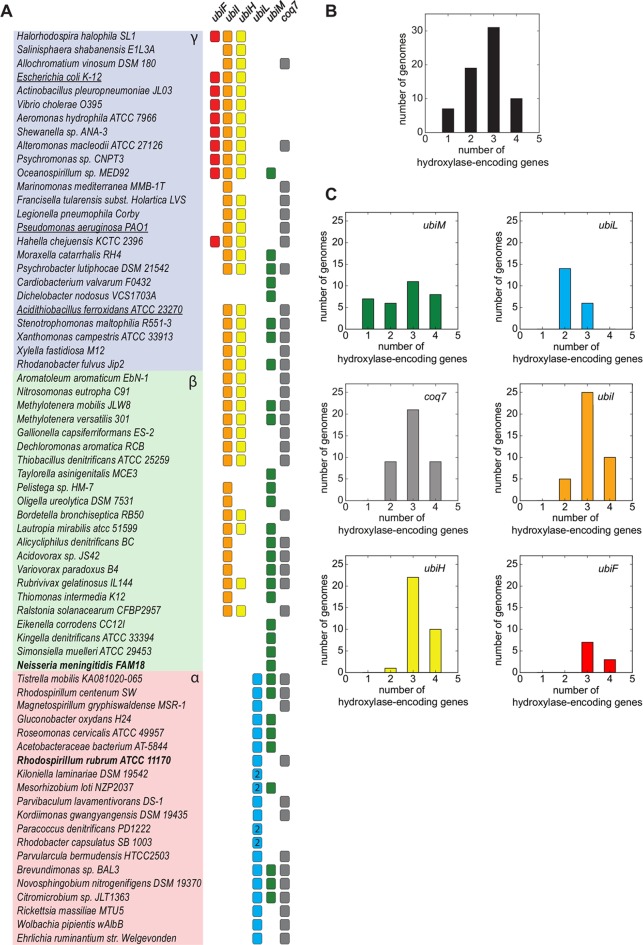
Distribution of the known and new candidate genes involved in hydroxylation reactions of UQ biosynthesis in proteobacteria. (A) The occurrence among alpha-, beta- and gammaproteobacteria (in pink, green, and blue, respectively) of known UQ hydroxylase-encoding genes (*ubiF*, *ubiH*, *ubiI*, and *coq7*) and potential new candidates (*ubiL* and *ubiM*) is indicated by colored squares in the corresponding columns. Columns are left blank when genes are absent. Paralogy is designated with the numbers of gene copies found in given genomes. Species whose UQ hydroxylases have been characterized previously and in our study are underlined and in boldface, respectively. Note the wide distribution of *ubiH*, *ubiI*, *ubiM*, and *coq7* compared to that of *ubiF* and *ubiL*. The identification numbers of the corresponding proteins are given in Table S1 in the supplemental material. (B) Numbers of genomes containing 1, 2, 3, or 4 UQ hydroxylase-encoding genes. (C) Numbers of UQ hydroxylase-encoding genes in genomes containing a specific hydroxylase (for clarity and consistency, histogram colors are identical to box colors in panel A for each hydroxylase).

We investigated the taxonomic distribution of UQ hydroxylase-encoding genes in our 67 representative genomes of alpha-, beta-, and gammaproteobacteria (Fig. 3A). The *ubiF* and *ubiL* genes were found to be restricted to gamma- and alphaproteobacteria, respectively. *ubiH*, *ubiI*, *ubiM*, and *coq7* genes were more widely distributed. In four alphaproteobacterial genomes, two copies of *ubiL* were present; for example, in the genome of *Paracoccus denitrificans* strain PD1222 (Fig. 3A). Overall, we observed 19 different combinations of the five FMO- and Coq7-encoding genes, thereby extending tremendously our view of the bacterial hydroxylation systems used for UQ biosynthesis (see Table S2 in the supplemental material). Most genomes contained three UQ hydroxylase-encoding genes, with noticeable exceptions containing only two or even a single gene (Fig. 3B). The latter cases suggested either that the corresponding UQ hydroxylases may hydroxylate more than one position of the aromatic ring (i.e., they may have a broad regioselectivity) or that other UQ hydroxylases have yet to be identified in these genomes. Interestingly, *ubiF*, *ubiH*, *ubiI*, and *coq7* were mostly found in genomes containing three hydroxylase-encoding genes (Fig. 3C). In contrast, UbiL was typically associated with only one other UQ hydroxylase and UbiM was found in combination with either zero, one, two, or three hydroxylases in a comparable number of genomes (Fig. 3C). Altogether, these data thus suggest that UbiM and UbiL may frequently feature a broad regioselectivity. To validate this hypothesis, we performed functional studies of the UQ hydroxylases from *Rhodospirillum rubrum* (one UbiL and one Coq7 homolog) and *Neisseria meningitidis* (a single UbiM homolog) (Fig. 3A).

10.1128/mSystems.00091-16.8 Table S2  Distribution of the different combinations of UQ hydroxylase-encoding genes within the orders of alpha-, beta- and gamma-proteobacteria. + and ++ indicate the presence of one and two copy(ies) of the corresponding gene, respectively. Download Table S2, PDF file, 0.1 MB. Copyright © 2016 Pelosi et al. 2016 Pelosi et al. This content is distributed under the terms of the Creative Commons Attribution 4.0 International license.

### UbiL and Coq7 from *R. rubrum* respectively complement C-5/C-1 and C-6 hydroxylation defects in *E. coli*.

**FIG 4:**
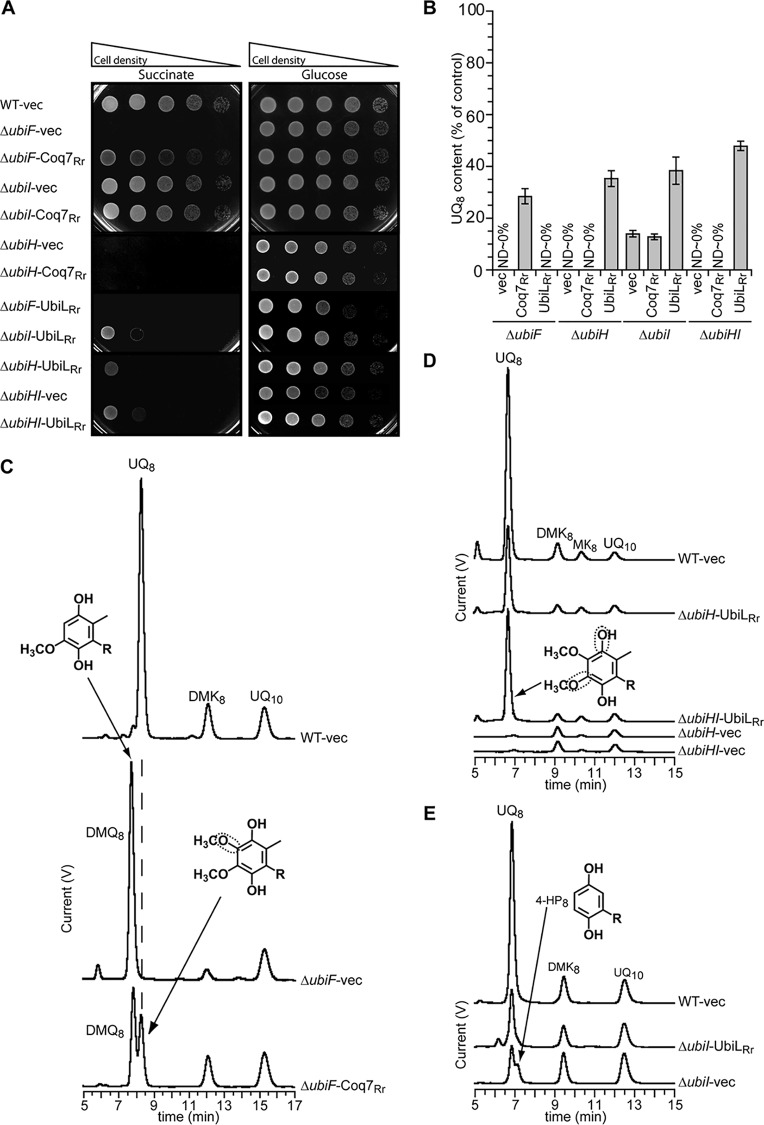
Complementation analyses of *E. coli* UQ biosynthesis mutants using hydroxylases from *R. rubrum* (indicated by “Rr”). (A) Δ*ubiF*, Δ*ubiI*, Δ*ubiH*, and Δ*ubiHI E. coli* mutant strains transformed with the pTrc99a empty vector (vec), the pTrc99a-*coq7_Rr_* vector encoding Coq7_Rr_, or the pTrc99a-*ubiL_Rr_* vector encoding UbiL_Rr_ were grown in LB for 4 h at 37°C. The *E. coli* wild-type strain MG1655 (WT) transformed with the pTrc99a empty vector was used as a control. Serial dilutions were spotted onto plates containing M9 minimal medium with 0.4% (wt/vol) either glucose or succinate as the sole carbon source. The plates were incubated overnight at 37°C. (B) Quantification of cellular UQ_8_ contents of the *E. coli* strains grown in LB medium, expressed as the percentages of the control value, which corresponds to the UQ_8_ content of the wild-type strain harboring pTrc99a (*n* = 3). ND, not detected. Error bars show standard deviations. (C, D, and E) HPLC-ECD analysis of lipid extracts from 1-mg amounts of cells after growth in LB medium. The chromatograms are representative of 3 independent experiments. The peaks corresponding to UQ_8_, C-6-demethoxy-ubiquinone (DMQ_8_), demethylmenaquinone (DMK_8_), menaquinone (MK_8_), 3-octaprenyl-4-hydroxyphenol (4-HP_8_), and the UQ_10_ standard are indicated. The framed C-O bonds on the UQ molecules (R, octaprenyl tail) depict the positions hydroxylated by the heterologous enzyme.

*E. coli* Δ*ubiH* and Δ*ubiF* mutant strains are unable to synthesize UQ_8_ under aerobic conditions and are thus unable to grow on a respiratory medium containing succinate (Fig. 4A) (20). In contrast, their growth on a fermentative medium with glucose is not severely affected (Fig. 4A) (20). We have previously shown that *E. coli* Δ*ubiI* cells accumulated 3-octaprenyl-4-hydroxyphenol (4-HP_8_) and had a highly decreased level of UQ_8_ (11), which was, however, sufficient to support growth on succinate (Fig. 4A; see also Fig. S3A in the supplemental material). The residual UQ_8_ content of Δ*ubiI* cells (~10% of the level in wild-type [WT] cells) is due to the limited hydroxylation of position C-5 by UbiF (11).

10.1128/mSystems.00091-16.3 Figure S3  Coq7_Rr_ and UbiL_Rr_ do not catalyze C-5/C-1 and C-6 hydroxylation in *E. coli*, respectively. Bacteria were grown in LB for 4 h at 37°C. HPLC-ECD analysis of lipid extracts from 1-mg amounts of the *E. coli* Δ*ubiI* (A) and Δ*ubiH* (B) mutant strains transformed with an empty vector (vec) or pTrc99a-*coq7_Rr_* vector encoding Coq7_Rr_. (C) HPLC-ECD analysis of lipid extracts from 1-mg amounts of the *E. coli* Δ*ubiF* mutant strain transformed with an empty vector (vec) or pTrc99a-*ubiL_Rr_* vector encoding UbiL_Rr_. The *E. coli* wild-type strain MG1655 (WT) transformed with an empty vector was used as a control. The peaks corresponding to UQ_8_, 6-demethoxy-ubiquinone (DMQ_8_), demethylmenaquinone (DMK_8_), 3-octaprenyl-4-hydroxyphenol (4-HP_8_), and the UQ_10_ standard are indicated. Download Figure S3, PDF file, 0.1 MB. Copyright © 2016 Pelosi et al. 2016 Pelosi et al. This content is distributed under the terms of the Creative Commons Attribution 4.0 International license.

To test whether the proteins that we identified as potential UQ hydroxylases in *R. rubrum* were indeed involved in UQ biosynthesis, we verified their capacity to functionally complement *E. coli* strains in which the UQ hydroxylase-encoding genes were inactivated. The expression of Coq7 from *R. rubrum* (hereinafter called Coq7_Rr_) restored the growth of *E. coli* Δ*ubiF* cells in minimal medium containing succinate, suggesting a C-6 hydroxylase activity of Coq7_Rr_ (Fig. 4A). Coq7_Rr_, however, was unable to complement the growth defect of Δ*ubiH* cells and did not affect the growth of Δ*ubiI* cells (Fig. 4A). The cellular contents of isoprenoid quinones detected by high-performance liquid chromatography (HPLC)-electrochemical detection (ECD) of lipid extracts were in agreement with the observed phenotypes: Coq7_Rr_ restored UQ_8_ biosynthesis in Δ*ubiF* cells to 28% of the WT level (Fig. 4B) and concomitantly decreased the accumulation of C-6-demethoxy-ubiquinone (DMQ_8_), which is the substrate of UbiF (Fig. 4C). The quinone contents of Δ*ubiH* and Δ*ubiI* cells were not affected by the presence of the *coq7_Rr_* gene (Fig. 4B; see also Fig. S3A and S3B in the supplemental material). Overall, Coq7_Rr_ complemented the UQ_8_ biosynthesis defect only in Δ*ubiF* cells, thereby showing that Coq7_Rr_ functions exclusively as a C-6 hydroxylase when expressed in *E. coli*. The regioselectivity of this alphaproteobacterial Coq7 is similar to that of the two gammaproteobacterial Coq7 proteins previously studied (16).

When compared to *E. coli* UQ FMOs, the *R. rubrum* UbiL homolog (UbiL_Rr_) displayed 34 to 38% sequence identity. Nevertheless, UbiL_Rr_ was unable to restore the growth of the *E. coli* Δ*ubiF* mutant in minimal succinate medium (Fig. 4A), which was associated with the absence of UQ_8_ in Δ*ubiF* cells harboring *ubiL_Rr_* (Fig. 4B; see also Fig. S3C in the supplemental material). In contrast, UbiL_Rr_ partially rescued the growth of *E. coli* Δ*ubiH* and Δ*ubiHI* cells in minimal succinate medium. However, unexpectedly, UbiL_Rr_ negatively altered the growth of the Δ*ubiI* strain (Fig. 4A). The levels of UQ_8_ in Δ*ubiH* and Δ*ubiHI* cells were increased in the presence of UbiL_Rr_ to ~35 and 48% of the WT content, respectively (Fig. 4B and D). UbiL_Rr_ also increased the UQ_8_ content of the Δ*ubiI* strain (Fig. 4B) and abolished the accumulation of 4-HP_8_, which is produced as a consequence of the C-5 hydroxylation defect (Fig. 4E). These results demonstrate that UbiL_Rr_ is able to efficiently hydroxylate both the C-1 and C-5 positions when expressed in *E. coli*. Altogether, our phylogenetic and biochemical data support the idea that *R. rubrum* utilizes only two hydroxylases to synthesize UQ: Coq7_Rr_ with specificity for position C-6 and UbiL_Rr_ with a broader regioselectivity for both C-1 and C-5.

### UbiM from *N. meningitidis* complements C-1/C-5/C-6 hydroxylation defects in *E. coli*.

**FIG 5:**
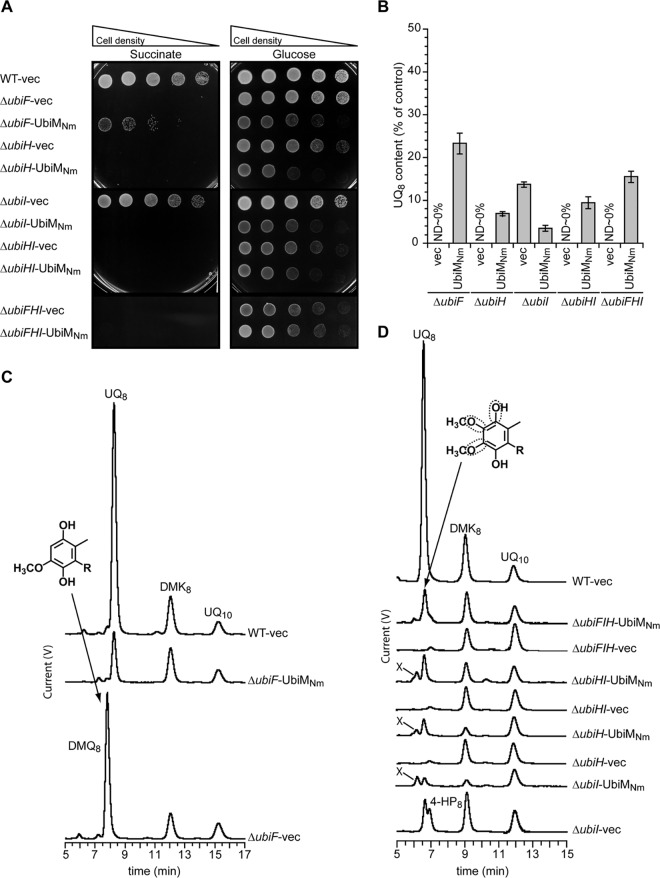
Complementation analyses of *E. coli* UQ biosynthesis mutants using the hydroxylase from *N. meningitidis*. (A) Δ*ubiF*, Δ*ubiI*, Δ*ubiH*, Δ*ubiHI*, and Δ*ubiFHI E. coli* mutant strains transformed with pTrc99a (vec) or with pTrc99a-*ubiM_Nm_* were grown in LB for 4 h at 37°C. The *E. coli* wild-type strain MG1655 (WT) transformed with pTrc99a was used as a control. Serial dilutions were spotted onto plates containing M9 minimal medium with 0.4% (wt/vol) either glucose or succinate as the sole carbon source. The plates were incubated overnight at 37°C. (B) Quantification of cellular UQ_8_ contents of *E. coli* strains grown in LB medium, expressed as percentages of the control value, which corresponds to the UQ_8_ content of the wild-type strain harboring pTrc99a (*n* = 3). ND, not detected. Error bars show standard deviations. (C and D) HPLC-ECD analysis of lipid extracts from 1-mg amounts of cells after growth in LB medium. The chromatograms are representative of 3 independent experiments. The peaks corresponding to UQ_8_, C-6-demethoxy-ubiquinone (DMQ_8_), demethylmenaquinone (DMK_8_), menaquinone (MK_8_), 3-octaprenyl-4-hydroxyphenol (4-HP_8_), and the UQ_10_ standard are indicated. The framed C-O bonds on the UQ molecules (R, octaprenyl tail) depict the positions hydroxylated by the heterologous enzyme.

Our phylogenetic analysis revealed that *N. meningitidis* does not contain *ubiH*, *ubiF*, *ubiI*, *ubiL*, or *coq7* but possesses instead a single UQ hydroxylase-encoding gene, *ubiM*_Nm_ (Fig. 3A). We therefore hypothesized that UbiM_Nm_, which shares 24 to 28% identity with *E. coli* UQ FMOs, may catalyze hydroxylation reactions at all three positions, C-1, C-5, and C-6, in UQ biosynthesis in *N. meningitidis*. By expressing UbiM_Nm_ in *E. coli* mutants, we found that UbiM_Nm_ partially restored the growth of the *E. coli* Δ*ubiF* strain in minimal succinate medium (Fig. 5A). In contrast, UbiM_Nm_ was unable to restore the growth of the *E. coli* Δ*ubiH*, Δ*ubiHI*, and Δ*ubiFHI* strains in minimal succinate medium and was even detrimental for Δ*ubiI* cells (Fig. 5A). The expression of UbiM_Nm_ also delayed growth in minimal glucose medium (except for the *E. coli* Δ*ubiFHI* strain), suggesting a toxic effect unrelated to respiration.

**FIG 6:**
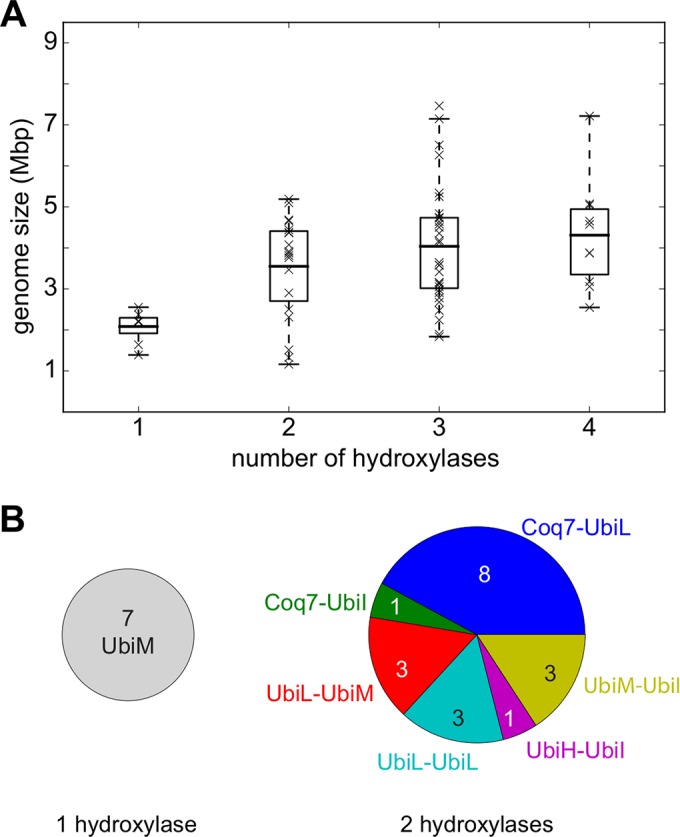
Relationship between genome size and hydroxylase content. (A) Sizes of genomes and numbers of hydroxylases they contain, revealing a significant correlation (correlation coefficient, *r* ≃ 0.40, *P* ≃ 8.10^−4^; for the hypothesis the slope is zero). Boxes extend from the lower to the upper quartile values of the data; whiskers indicate an extra half quartile on each side of them. The horizontal line in each box indicates the mean value of the data. (B) Pie charts showing the combinations of hydroxylases for the genomes containing one and two hydroxylases. The seven small genomes with one hydroxylase (left) contain UbiM exclusively (see Fig. 3 also), while the majority of genomes with two hydroxylases (right) contain at least one copy of UbiL.

**FIG 7:**
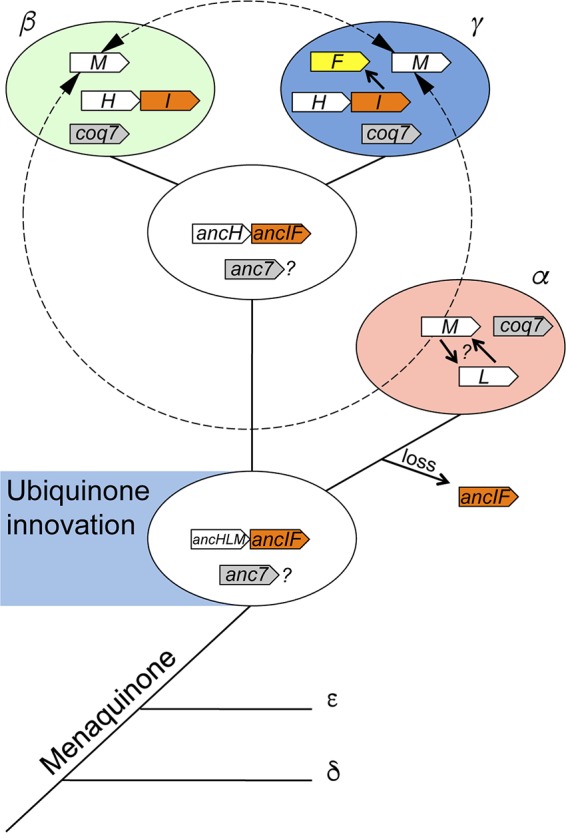
Evolutionary dynamics of UQ FMOs in the proteobacterial lineage. The schematic phylogenetic tree is based on 16S rRNA sequences of the proteobacterial subclasses. The potential innovation of the UQ molecule in the alpha-, beta-, and gammaproteobacterial common ancestor is indicated. The different UQ FMO-encoding genes are represented within the three proteobacterial subclasses and their common ancestors. “*anc*” stands for the ancestral forms of the respective genes. The duplication of *ubiI* that gave rise to *ubiF* and the duplication that occurred for *ubiM* and *ubiL* are indicated by solid arrows. Potential horizontal gene transfer events are shown with dashed-line arrows.

We next assayed the cellular UQ_8_ content of each strain. In agreement with the growth in minimal succinate medium, UbiM_Nm_ rescued UQ_8_ biosynthesis in *E. coli* Δ*ubiF* to 23% of the amount present in the WT strain (Fig. 5B) and strongly decreased the accumulation of DMQ_8_ (Fig. 5C), which is formed as a result of a C-6 hydroxylation defect. UbiM_Nm_ also restored the biosynthesis of UQ_8_ in the *E. coli* Δ*ubiH*, Δ*ubiHI*, and Δ*ubiFHI* strains (Fig. 5D) to 7, 10, and 16% of the reference UQ_8_ contents, respectively (Fig. 5B). In contrast, UQ_8_ biosynthesis in the *E. coli* Δ*ubiI* strain was decreased by about fourfold in the presence of UbiM_Nm_ (Fig. 5B and D), consistent with the observed growth phenotype (Fig. 5A).

An unknown redox-active compound (compound X) was observed when UbiM_Nm_ was expressed in the *E. coli* Δ*ubiI*, Δ*ubiH*, and Δ*ubiHI* strains (Fig. 5D). Analysis by mass spectrometry (MS) in positive mode gave *m*/*z* ratios (M + NH_4_^+^) of 730.2 for the oxidized compound X and 732.2 for its reduced form (see Fig. S4A and B in the supplemental material), consistent with a ubiquinol ring lacking a methyl group and functionalized with an octaprenyl tail (i.e., C-2-demethyl-UQ_8_, O_5_-demethyl-UQ_8_, or O_6_-demethyl-UQ_8_) (see Fig. S4C). The accumulation of this compound may interfere with the function of UQ_8_ in the respiratory chain and, thus, prevent the growth of UQ_8_-producing strains in minimal succinate medium (Fig. 5A). Nevertheless, our results unambiguously demonstrate that UbiM_Nm_ catalyzes hydroxylation reactions at positions C-1, C-5, and C-6 when expressed in *E. coli*, uncovering a broad regioselectivity that is unprecedented among UQ hydroxylases. Our results strongly suggest that UbiM_Nm_ performs all three hydroxylation reactions of the UQ biosynthetic pathway in *N. meningitidis*, consistent with our phylogenetic identification of a single UQ hydroxylase-encoding gene in this genome.

10.1128/mSystems.00091-16.4 Figure S4  Compound X synthesized in *E. coli* cells expressing UbiM_Nm_. (A and B) HPLC-mass spectrometry analysis of compound X in lipid extracts from Δ*ubiI* cells expressing UbiM_Nm_ with the precolumn electrode set in oxidizing (+650 mV) (A) or reducing (−700 mV) (B) mode. (C) Potential structures and names of compound X. Download Figure S4, PDF file, 0.2 MB. Copyright © 2016 Pelosi et al. 2016 Pelosi et al. This content is distributed under the terms of the Creative Commons Attribution 4.0 International license.

### Distribution of UQ hydroxylase-encoding genes and genome sizes.

Given that one to four UQ hydroxylases are found across our representative UQ-producing proteobacteria (Fig. 3B), we asked whether the number of UQ hydroxylase genes might correlate with the genome size. We indeed found a significant positive correlation, with a coefficient of *r* ≃ 0.40 (*P* ≃ 8.10^−4^; for the hypothesis the slope is zero) (Fig. 6A). In particular, compared to the wide range of sizes observed for the proteobacterial genomes (Fig. 6A; see also Fig. S5A in the supplemental material), the seven genomes containing a single UQ hydroxylase-encoding gene are all small, less than 2.6 Mbp (*P* ≃ 10^−8^, one-sided *t* test with unequal variances). UbiM was the unique UQ hydroxylase found in these seven genomes (Fig. 6B and 3), suggesting that the presence of UbiM alone may be related to genome reduction. Along the same line, most genomes (14 of 19) with two UQ hydroxylase-encoding genes contain at least one copy of *ubiL* (Fig. 6B), suggesting that the broad regioselectivity of UbiL may accommodate genome reduction. Nevertheless, our analysis shows that a few genomes of less than 2.6 Mbp have three or four hydroxylase-encoding genes (Fig. 6A). Moreover, the average mean genome sizes of proteobacteria containing a given type of UQ hydroxylase were comparable across all types of UQ hydroxylases (see Fig. S5B). Altogether, our results show that *ubiL* and *ubiM* are present in small genomes more often than the other UQ hydroxylase-encoding genes and that *ubiL* and *ubiM* are also distributed among large genomes.

10.1128/mSystems.00091-16.5 Figure S5  Genome sizes of proteobacteria, UQ hydroxylase-encoding genes, and phylogenetic classes. (A) Genome size distribution and phylogenetic class (alpha, beta, and gammaproteobacteria). The middle line in each boxplot indicates the mean value. The whiskers indicate 1.5× interquartile range (IQR). Data points outside the whiskers are considered outliers. (B) Genome size distribution according to specific UQ hydroxylases, showing comparable mean values. Download Figure S5, PDF file, 0.1 MB. Copyright © 2016 Pelosi et al. 2016 Pelosi et al. This content is distributed under the terms of the Creative Commons Attribution 4.0 International license.

## DISCUSSION

### Unsuspected diversity in the UQ hydroxylase repertoire.

Our phylogenetic investigation revealed that proteobacteria evolved a surprising variety of enzymatic combinations to hydroxylate the three contiguous positions of the aromatic ring of UQ. Prior to our study, four UQ hydroxylases (the di-iron monooxygenase Coq7 and the three FMOs UbiF, UbiH, and UbiI) had been characterized, constituting two different protein sets in *E. coli* (UbiF, UbiH, and UbiI) (11) and in *P. aeruginosa* and *A. ferrooxidans* (UbiH, UbiI, and Coq7) (16). Here, we identified two new clades of UQ FMOs (UbiM and UbiL) and found a total of 19 combinations of the six hydroxylases in proteobacteria.

The hydroxylase repertoire is highly diverse at the subclass level, with at least nine different combinations for gammaproteobacteria, and even at the taxonomic level, with, for example, five different combinations within the *Burkhoderiales* and three within the *Oceanospirillales*, *Pseudomonadales*, and *Rhodobacterales* (see Table S2 in the supplemental material). The hydroxylase combination that includes UbiH, UbiI, and Coq7 is the most widespread, being present in 11 orders (Fig. 3A). Intriguingly, some beta- and gammaproteobacteria contain four UQ hydroxylase-encoding genes (Fig. 3A and B). In such cases, it is difficult to speculate about the specific function of each UQ hydroxylase; some may be redundant or differentially expressed under various growth conditions. We think it unlikely that organisms with four UQ hydroxylase-encoding genes might use a precursor of UQ requiring four hydroxylation reactions rather than 4-HB, which requires three (Fig. 1). Indeed, *Xanthomonas campestris* carries four UQ hydroxylase-encoding genes (Fig. 3A), and yet, this organism has recently been shown to use 4-HB to synthesize UQ (21). Of interest, 4-HB is synthesized in *X. campestris* by the XanB2 protein (Fig. 1), a bifunctional chorismatase unrelated to UbiC (21).

### Scenario for the emergence and distribution of the UQ hydroxylases.

The innovation of UQ likely occurred in the common ancestor of the alpha-, beta-, and gammaproteobacteria (5). Based on our results, we propose a parsimonious evolutionary scenario with vertical descent and horizontal gene transfer contributing to the distribution of UQ hydroxylases in alpha-, beta-, and gammaproteobacteria (Fig. 7). The location of the root represented by the outgroup of FMOs on the phylogenetic tree (Fig. 2) indicates the existence of two ancestral genes that gave rise to the UbiH/UbiL/UbiM and the UbiI/UbiF proteins (referred to hereinafter as *ancHLM* and *ancIF*, respectively) (Fig. 7). On the left side of the root, a striking picture of the exclusive allocations of UbiH in beta- and gammaproteobacteria and UbiL in alphaproteobacteria can be observed. The overall topology of the UbiH, UbiL, and UbiM clades in conjunction with the taxonomic origins of sequences depicts the ribosomal tree of life (22) and designates the root as the common ancestor of alpha-/beta-/gammaproteobacterial subclasses.

On one hand, AncHLM evolved into AncH, an ancestral UbiH protein in the common ancestor of beta- and gammaproteobacteria, as supported by the well-defined split between the UbiH sequences from these two proteobacterial subclasses (Fig. 2). On the other hand, AncHLM evolved into UbiL in alphaproteobacteria. The branching of UbiM and UbiL protein sequences suggests a duplication event that may have occurred in the alphaproteobacterial ancestor (Fig. 7). The subsequent dissemination of UbiM to the other proteobacterial subclasses may be explained by horizontal gene transfer and the selective benefit conferred by its versatile regioselectivity, which has probably been conserved, as suggested by the short branches that characterize this clade.

The presence of UbiI only in beta- and gammaproteobacteria suggested that AncIF was present in the common ancestor of beta- and gammaproteobacteria and was lost by the alphaproteobacterial ancestor. The loss of the *ancIF* gene may have been favored by the broadening of UbiL regioselectivity. Since *ubiF* is only found in 40% of the gammaproteobacterial species inspected and always in genomes also encoding *ubiI*, we suspect that *ubiF* originated from a duplication event involving *ubiI* within the gammaproteobacteria. Our suggestion that *ubiF* evolved later than *ubiI* is supported by the conservation of the tandem association of *ubiI* and *ubiH* within the beta- and gammaproteobacterial subclasses.

The branching of the eukaryotic Coq6 sequences within the gammaproteobacterial sequences is puzzling given the current theory that relates the mitochondrial endosymbiotic event to the alphaproteobacterial subclass (23). In our opinion, this result may be interpreted in either of two ways: (i) the early loss of AncIF from alphaproteobacteria might have occurred after the endosymbiotic event and the consequent lack of a UbiI alphaproteobacterial phylogenetic signal led the algorithms to construct an artifactual topology or (ii) Coq6 might be derived from a lateral gene transfer event from the gammaproteobacteria that occurred after the mitochondrial endosymbiotic event.

It is noteworthy that *coq7* genes are found in all three subclasses, alpha-, beta-, and gammaproteobacteria, and an ancestral *coq7* gene might therefore have been present in their common ancestor. Unfortunately, the short size of Coq7 primary sequences conveys insufficient phylogenetic information to allow confident analysis with the phylogenetic reconstruction tools available.

### Molecular basis of the regioselectivity of UQ FMOs.

All UQ FMOs belong to the class A flavin monooxygenases and utilize a similar chemistry for hydroxylation, with the formation of a reactive flavin-hydroperoxide which transfers a hydroxyl group onto the substrate (12). The position of the carbon atom of the substrate that is hydroxylated by a given UQ FMO is dictated by the orientation of the substrate with respect to the flavin-hydroperoxide within the enzyme’s active site (24, 25). A large number of amino acids may contribute to the positioning of the substrate in UQ FMOs, since these proteins are expected to contain a large cavity to accommodate the long, hydrophobic polyisoprenoid chain of the substrate. This assumption is supported by a recent homology model of the yeast (*S. cerevisiae*) Coq6 protein, which showed the substrate to be located within a long tunnel (26). The only crystal structure available for a UQ FMO is that of a truncated form of UbiI that does not contain its flavin cofactor (11) and, thus, provides limited information about the structure of the active site. Therefore, it is currently impossible to identify which residues are involved in the regioselectivities of the different UQ FMOs. Our work further illustrates the interesting possibilities offered by the UQ FMO family for structure-function studies and should prompt new investigations.

### Differing regioselectivities among and within UQ hydroxylase clades.

We demonstrated broad regioselectivity for UbiM from *N. meningitidis* and UbiL from *R. rubrum*, since they are able to hydroxylate 2 and 3 positions of the UQ head group, respectively. The high representation of *ubiM* and *ubiL* genes in genomes that contain fewer than three UQ hydroxylases (Fig. 3C) supports the idea that the capacity to hydroxylate multiple positions is a hallmark of the UbiM and UbiL clades. However, we expect the regioselectivities of UbiM and UbiL to vary in different microorganisms. Indeed, UbiM is present in combination with other UQ hydroxylases in many organisms (Fig. 3), suggesting that UbiM proteins from these bacteria may have narrower regioselectivities than UbiM_Nm_. We also found that *Kiloniella laminariae*, *Paracoccus denitrificans*, and *Rhodobacter capsulatus* possess only two genes encoding UbiL (Fig. 3A). Therefore, it is highly likely that these combinations of two UbiL proteins are able to hydroxylate all three positions of the UQ head group, as opposed to UbiL_Rr_, which only hydroxylated positions C-1 and C-5 (Fig. 4D).

In contrast to UbiM and UbiL, our observation that UbiF, UbiH, UbiI, and Coq7 are mostly found in genomes containing three hydroxylase-encoding genes (Fig. 3C) suggests that these proteins are generally hydroxylating a single position of the aromatic ring. In the case of Coq7 and UbiF, a preference for position C-6 is likely the norm, given the following lines of evidence. (i) Coq7 proteins from two gammaproteobacteria have been shown to be specific for position C-6 (16), and we have extended this trait to the alphaproteobacterial Coq7_Rr_ protein (Fig. 4; see also Fig. S3 in the supplemental material). (ii) *E. coli* UbiF has been shown to hydroxylate C-6 (20). (iii) Both Coq7 and UbiF also co-occur with UbiH and UbiI, which in *E. coli* have preferences for C-1 and C-5, respectively (11, 20), and we expect the regioselectivities of UbiH and UbiI to be conserved in most beta- and gammaproteobacterial proteins, given their strong co-occurrence properties (Fig. S6). Finally, (iv) Coq7 and UbiF tend to be present in different genomes, as revealed by their strong anticorrelation for co-occurrence properties (see Fig. S6), which is likely to be the consequence of both enzymes hydroxylating the same position.

10.1128/mSystems.00091-16.6 Figure S6  Co-occurrence (C) of the UQ hydroxylase-encoding genes present in the beta- and gammaproteobacteria. C is equal to the Pearson correlation of the corresponding presence/absence profiles (also known as the phylogenetic profiles) of two given genes. Each pixel indicates the tendency of two genes to co-occur (C > 0, in red) or not (C < 0, in blue) in different genomes, with the intensity of color being proportional to the strength of that tendency. UbiF and UbiI function as a pair that is mostly associated with either Coq7 or UbiF but not with both enzymes together. UbiM tends to be present alone. Download Figure S6, PDF file, 0.1 MB. Copyright © 2016 Pelosi et al. 2016 Pelosi et al. This content is distributed under the terms of the Creative Commons Attribution 4.0 International license.

Overall, distinct regioselectivities may be associated with the different UQ hydroxylase clades. However, the 19 UQ hydroxylase combinations that we identified suggest that regioselectivity is not absolute within the UQ hydroxylase clades and that variations have evolved across proteobacterial species to meet the requirement of hydroxylating three positions of the UQ head group. Such a diversity in regioselectivities is, to our knowledge, unique among FMOs. Hydroxylases distinct from FMOs, such as ring-hydroxylating dioxygenases (RHOs) and the polysaccharide monooxygenases (PMOs), have also recently been shown to preferentially hydroxylate particular substrate positions according to their clades (27–30).

### Evolution of generalist enzymes within the UbiL and UbiM clades.

Our phylogenetic and biochemical analyses revealed that members of the UbiM and UbiL clades exhibit broad regioselectivities and may therefore be considered generalist enzymes. In contrast, Coq7 proteins and members of the UbiH and UbiIF clades may be specialist enzymes, given their restricted regioselectivities.

Why was the emergence of generalist enzymes limited to the UbiM and UbiL clades? Protein evolution is rarely the result of a single mutation and often involves mutations that do not alter the protein function but open evolutionary paths that subsequently yield innovations, such as the regioselectivity here (31). Thus, the ancestral regioselective UbiM and UbiL proteins may have been fewer mutational steps away from broad regioselectivity than UbiI, UbiH, or UbiF.

One unexpected finding of our study is the variability in the numbers of UQ hydroxylases (one to four) among proteobacteria. Interestingly, the numbers of UQ hydroxylases were found to correlate with the sizes of genomes (Fig. 6A). This suggests that organisms with smaller genomes may have favored the loss of genes encoding proteins in the UQ hydroxylase family. The evolution of generalist proteins produced a functional overlap with specialist UQ hydroxylases and, thus, may have allowed the loss of specialist UQ hydroxylase-encoding genes without compromising UQ biosynthesis. This hypothesis is consistent with the concept that bacteria with reduced genomes tend to maintain the number of protein families at the expense of family size, resulting in protein families with a single gene representative (32).

### Potential limitations to the evolution of generalist UQ hydroxylases.

Most proteobacteria have maintained three hydroxylases to synthesize UQ, whereas others can fulfill a similar function with a single generalist enzyme (Fig. 3B). This suggests that the evolution of a single generalist UQ hydroxylase may be detrimental in some situations. Indeed, it will likely result in the production of new UQ biosynthetic intermediates that may cause toxicity or alter the recognition by other Ubi proteins. Indeed, UbiM_Nm_ is likely to hydroxylate all three positions, C-1, C-5, and C-6, at once and, thus, release a product corresponding to a prenylated aromatic ring with four contiguous hydroxyl groups. This product is not synthesized in *E. coli* because the regioselective hydroxylation reactions catalyzed by UbiI and UbiF are immediately followed by O-methylation reactions by UbiG (Fig. 1).

A UQ intermediate with multiple contiguous hydroxyl groups, as formed by UbiM_Nm_, may be harmful for cells, since a chemically similar compound, 1,2,3,4-tetrahydroxybenzene, was reported to exhibit antimicrobial activity (33). Besides toxicity, the new UQ biosynthetic intermediates may not be appropriate substrates for downstream enzymes of the pathway. We indeed noticed that *E. coli* strains expressing UbiM_Nm_ produced compound X, which lacks a methyl group compared to UQ (Fig. 5; see also Fig. S4 in the supplemental material). Thus, one methylation reaction is impaired by the heterologous expression of UbiM_Nm_. This likely reflects the inability of either the C methyltransferase UbiE or the O methyltransferase UbiG to efficiently methylate the multihydroxylated UQ intermediate produced by the generalist UbiM_Nm_ protein. Therefore, evolution of UbiE or UbiG may be required to accommodate new UQ biosynthetic intermediates resulting from the emergence of generalist UQ hydroxylases. Such a requirement for further evolution of partner Ubi enzymes may thus limit the emergence of generalist UQ hydroxylases.

### Conclusion.

Here, we showed that proteobacteria have evolved an unsuspected variety of combinations to hydroxylate the three contiguous positions of the head group of UQ. The demonstration that related UQ FMOs differ in their capacity to hydroxylate one or several sites of a common substrate paves the way for structural and functional studies to understand the control of regioselectivity within the different clades of UQ FMOs. Our results revisit the current paradigm for the requirement of three distinct hydroxylases for UQ biosynthesis, highlight different potential evolutionary trajectories toward generalist enzymes, and contribute to the understanding of the innovation of UQ.

## MATERIALS AND METHODS

### Phylogenetic analyses.

The genomes used in this study were selected in two steps. First, we initially included one complete genome per order and two scaffold genomes for the *Kiloniellales* and *Kordiimonadales*. The phylogenetic analysis of UQ FMOs from these genomes revealed the existence of two new clades, UbiL and UbiM. At this point of the investigation (January 2014), we decided to include more genomes in order to (i) populate the UbiM and UbiL clades in an exhaustive way and (ii) explore more thoroughly the different combinations of UQ hydroxylases, which turned out to be more variable than expected (see Table S2 in the supplemental material). Details about our final set of 67 representative genomes can be found in Table S1.

Open reading frames (ORFs) encoding UQ FMOs were retrieved from the National Center for Biotechnology Information (NCBI; http://www.ncbi.nlm.nih.gov), using the UbiF (NP_415195), UbiH (NP_417383), and UbiI (NP_417382) sequences from *E. coli* K-12, the Coq7 sequence (NP_249346) from *P. aeruginosa*, and the Coq6 sequence from *Saccharomyces cerevisiae* (AAB61341) as query templates in BLAST searches with the server default parameters. Hits returned with a minimum score of e−10 were selected. Then, the corresponding protein sequences were aligned and a preliminary neighbor-joining tree (which also included our query sequences) was constructed to curate and sort the hits in categories defined by the tree topology. All accession numbers of the sequences used in this study can be found in Table S1 in the supplemental material.

The data sets for Coq6, FMOs of interest, and the outgroup were aligned individually using MUSCLE (34). Sites with ambiguous alignments were removed manually in Aliview software (35). The three data sets were then aligned together using the Profil alignment mode of ClustalX (36). Additional manual curation was performed to obtain the final multiple alignment, which encompassed 173 sequences and 474 positions. For the phylogenetic reconstruction, the evolutionary model was selected by a maximum-likelihood (ML) approach using Smart Model Selection (SMS) (37) and the Akaike information criterion. According to the results of the SMS analysis, the LG model (38) with a gamma-shaped distribution of rates across sites (LG+G6+F) was selected for the FMO global tree (LG, Le Gascuel; G6, gamma-shaped distribution with 6 substitution rate categories; F, frequencies of amino acid estimation). The ML trees were reconstructed using PhyML (37). Approximate likelihood ratio tests were performed to evaluate the robustness of the tree topologies (39). Bayesian inference was performed using MrBayes (40) with parameters (the substitution model, the gamma-shaped parameter, and the number of substitution categories; LG+G6+F) identical to those used by PhyML. The Markov chain Monte Carlo (MCMC) analysis was run with a temperature of 0.1 until evidence of proper mixing was obtained at 750,000 iterations. The eight chains were sampled every 250th iteration. The final average standard deviation of split frequencies was 0.032, the average ESS value calculated was >100, and the potential scale reduction factor was 1.000. The results were visualized and edited with FigTree 1.4.2 (http://tree.bio.ed.ac.uk/software/figtree/).

### Strain construction and growth.

All strains derive from *E. coli* K-12. Strains JW2874, JW2875, and JW0659 from the Keio Collection (41) were kindly provided by P. Moreau (LCB, Marseille) and were used as donors in conjugation experiments to construct the Δ*ubiI*::*kan*, Δ*ubiH*::*kan*, and Δ*ubiF*::*kan* mutant strains. The Δ*ubiI*, Δ*ubiH*, and Δ*ubiF* strains were cured with pCP20 to yield Δ*ubiIc*, Δ*ubiHc*, and Δ*ubiFc* strains*.* The Δ*ubiH* Δ*ubiI* double mutant was constructed as described previously (42). Briefly, the *ubiHI*::*cat* mutation was generated in a one-step inactivation of the *ubiHI* genes. A DNA fragment containing the *cat* gene flanked with a 5′ *ubiH* and a 3′ *ubiI* region was PCR amplified using pKD3 as a template and oligonucleotides 5′ wanner *ubiH* and 3′ wanner *ubiI* (see Table S3 in the supplemental material). Strain BW25113 carrying the pKD46 plasmid was transformed by electrotransformation with the linear PCR product, and selection for Cm^r^ clones was carried out. The resulting strain was used to transduce, using the P1 phage, the *ubiHI*::*cat* mutation into MG1655 or the Δ*ubiFc* strain, yielding Δ*ubiHI* and Δ*ubiFHI* strains, respectively. Mutations were confirmed by colony PCR with primers flanking the mutation. The strains used in this study are listed in Table S4.

10.1128/mSystems.00091-16.9 Table S3  Primers used in this study. Download Table S3, PDF file, 0.1 MB. Copyright © 2016 Pelosi et al. 2016 Pelosi et al. This content is distributed under the terms of the Creative Commons Attribution 4.0 International license.

10.1128/mSystems.00091-16.10 Table S4  *Escherichia coli* strains used in this study. Download Table S4, PDF file, 0.2 MB. Copyright © 2016 Pelosi et al. 2016 Pelosi et al. This content is distributed under the terms of the Creative Commons Attribution 4.0 International license.

*E. coli* strains were grown in lysogeny broth (LB)-rich medium or in M9 minimal medium (supplemented with glucose or succinate, 0.4% [wt/vol] final concentration) at 37°C. Ampicillin (100 µg/ml), kanamycin (50 µg/ml), and chloramphenicol (35 µg/ml) were added when needed.

### Cloning, plasmid construction, and complementation assays.

The *R. rubrum* and *N. meningitidis* genomic DNAs were generous gifts from John Willison (CEA, Grenoble, France) and Vivien Sutera (CHU, Grenoble, France), respectively. The ORFs encoding UbiL_Rr_ (NCBI accession number YP_428788), Coq7_Rr_ (YP_428579), and UbiM_Nm_ (EFM05404) were PCR amplified from the corresponding genomic DNA using Phusion high-fidelity polymerase (New England Biolabs) and specific primers (see Table S3 in the supplemental material). The PCR fragments were cloned into the pTRc99a vector at the *EcoRI*, *HindIII*, or *BamHI* site, and the cloning products were checked by sequencing. The plasmids were transformed into *E. coli* strains with mutation of the *ubiF*, *ubiI*, and *ubiH* genes (single, double or triple mutants), and complementation of the UQ_8_ biosynthetic defect was assessed by both measuring the quinone content and plating serial dilutions onto solid M9 minimal medium supplemented with glucose or succinate as the only carbon sources and overnight growth at 37°C.

### Analysis of the quinone content.

Quinone extraction and quantification by HPLC-ECD analysis were performed as previously described, except that ammonium acetate was used instead of lithium perchlorate (11). UQ_10_ was used as the standard, and a precolumn guard cell set at +650 mV allowed the quinones to be detected in their oxidized form. When mass spectrometry (MS) detection was needed, the flow was divided after the diode array detector with an adjustable split valve (Analytical Scientific Instruments) in order to allow simultaneous EC (60% of the flow) and MS (40% of the flow) detections. MS detection was achieved with an MSQ Plus spectrometer (Thermo Fisher), used in positive mode with electrospray ionization (24). The probe temperature was 450°C, the cone voltage was 80 V, and MS spectra were recorded between *m*/*z* 600 and 880 with a scan time of 0.4 s.

### Statistical analyses.

The co-occurrence between all possible pairs of hydroxylases from Coq7, UbiF, UbiH, UbiI, and UbiM was defined as the Pearson correlation of the corresponding presence/absence profiles obtained from genomes in beta- and gammaproteobacteria.
