# Supplementary material for: Evolution of Ubiquinone Biosynthesis: Multiple Proteobacterial Enzymes with Various Regioselectivities To Catalyze Three Contiguous Aromatic Hydroxylation Reactions
Source: mSystems. 2016 Aug 30;1(4):e00091-16. doi: 10.1128/mSystems.00091-16 (PMC5069965; doi:10.1128/mSystems.00091-16)
Supplement: Table S1 [file sys004162048st7.pdf]

Subclass/order	*Species*	Genome assembly status	Genome size	outgroup	UbiF	UbiH	UbiI	UbiL	UbiM	Coq7
**PROKARYOTES alpha-proteobacteria**										
Rhizobiales	*Parvibaculum lavamentivorans DS-1*	complete	3,91	ABS64776	-	-	-	WP_012110202	-	WP_012110542
	Mesorhizobium loti NZP2037	complete	7,46	WP_019856764<br>WP_027046882	-	-	-	WP_019861993<br>WP_019858090	WP_019856453	-
Rhodobacterales	*Rhodobacter capsulatus SB 1003*	complete	3,87	-	-	-	-	WP_013069027<br>WP_013066967	-	-
	Paracoccus denitrificans PD1222	complete	4,66	ABL71129	-	-	-	ABL70757<br>ABL72008	-	-
Rickettsiales	*Ehrlichia ruminantium str. Welgevonden*	complete	1,51	-	-	-	-	WP_011154692	-	WP_011154908
	Rickettsia massiliae MTU5	complete	1,33	-	-	-	-	WP_041404768	-	ABV84532
	Wolbachia pipientis wAlbB	complete	1,16	-	-	-	-	WP_006013756	-	CCE77271
Rhodospirilales	*Rhodospirillum rubrum ATCC 11170*	complete	4,38	-	-	-	-	YP_428788	-	YP_428579
	Rhodospirillum centenum SW	complete	4,36	-	-	-	-	ACJ00155	ACI98597	ACJ01458
	Acetobacteraceae bacterium AT-5844	scaffold	5,19	WP_007439601<br>WP_040290988<br>EHM01755<br>WP_007437232	-	-	-	WP_007437056	EHM01564	-
	Gluconobacter oxydans H24	complete	3,60	-	-	-	-	AFW01024	AFW01459	-
	Roseomonas cervicalis ATCC 49957	scaffold	5,10	EFH11333	-	-	-	EFH13112	EFH10994	-
	Tistrella mobilis KA081020-065	complete	3,92	-	-	-	-	AFK54975	AFK54292	WP_014746694
	Magnetospirillum gryphiswaldense MSR-1	complete	4,37	-	-	-	-	CAM74183	-	CAM75846
Sphingomonadales	*Novosphingobium nitrogenifigens DSM 19370*	scaffold	4.18	EGD57846	-	-	-	EGD57818	EGD59198	EGD60244
	Citromicrobium sp. JLT1363	contig	3,12	-	-	-	-	WP_010410420	WP_010412178	WP_010408913
Caulobacterales	*Brevundimonas sp. BAL3*	scaffold	3,65	-	-	-	-	WP_008260365	WP_008263583	WP_008261282
Kiloniellales	*Kiloniella laminariae DSM 19542*	scaffold	4,44	-	-	-	-	WP_020590378<br>WP_020592522	-	-
Kordiimonadales	*Kordiimonas gwangyangensis DSM 19435*	contig	4,08	WP_020398153	-	-	-	WP_020398587	-	WP_020398157
Parvularculales	*Parvularcula bermudensis HTCC2503*	complete	2,90	-	-	-	-	ADM08903	-	WP_013300890
**beta-proteobacteria**										
Neisseriales	*Neisseria meningitidis FAM18*	complete	2,19	-	-	-	-	-	EFM05404	-
	Simonsiella muelleri ATCC 29453	scaffold	2,37	-	-	-	-	-	EFG30291.2	-
	Kingella denitrificans ATCC 33394	scaffold	2,22	-	-	-	-	-	EGC16894	-
	Eikenella corrodens CC92I	scaffold	2,21	-	-	-	-	-	ETA83905	-
Burkholderiales	*Thiomonas intermedia K12*	complete	3,40	-	-	-	WP_013124173	-	WP_013121880	-
	Oligella ureolytica DSM 7531	scaffold	2,31	-	-	-	WP_018025923	-	WP_018025500	-
	Pelistega sp. HM-7	contig	2,50	-	-	-	WP_023951696	-	WP_023949944	-
	Alicycliphilus denitrificans BC	complete	4,64	-	-	-	WP_013520446	-	WP_013517013	WP_013517699
	Variovorax paradoxus B4	complete	5,80	AGU52002<br>AGU53625<br>AGU52348<br>AGU49355	-	-	WP_021012506	-	WP_021003848	WP_021005800
	Rubrivivax gelatinosus IL144	complete	5,04	-	-	WP_014430665	WP_014430352	-	WP_014428639	WP_014427025
	Acidovorax sp. JS42	complete	4,44	-	-	-	WP_011806715	-	WP_011803522	WP_011804133
	Taylorella asinigenitalis MCE3	complete	1,64	-	-	-	-	-	WP_014111363	-
	Lautropia mirabilis ATCC 51599	scaffold	3,15	-	-	EFV95960	EFV93749	-	WP_005674913	-
	Bordetella bronchiseptica RB50	chromosome	5,34	-	-	WP_003814217	WP_003815378	-	-	WP_003808447
	Ralstonia solanacearum CFBP2957	chromosome	3,42	CBJ53571	-	WP_013206950	WP_013205035	-	-	WP_013205072
Methylophilales	*Methylotenera mobilis JLW8*	complete	2,55	-	-	WP_015831538	WP_015831539	-	WP_012777491	WP_015831387
	Methylotenera versatilis 301	complete	3,06	-	-	WP_013147299	WP_013147300	-	WP_013148603	WP_013147126
Rhodocyclales	*Aromatoleum aromaticum EbN-1*	complete	4,73	-	-	WP_011236457	WP_011238131	-	-	WP_011236356
	Dechloromonas aromatica RCB	complete	4,50	-	-	WP_011289388	WP_011289235	-	-	WP_011286413
Gallionellales	*Gallionella capsiferriformans ES-2*	complete	3,16	-	-	WP_013292129	WP_013294578	-	-	WP_013292416
Nitrosomonadales	*Nitrosomonas eutropha C91*	complete	2,78	-	-	WP_011634282	WP_011634736	-	-	WP_011633566
Hydrogenophilales	*Thiobacillus denitrificans ATCC 25259*	complete	2,91	-	-	WP_011312845	WP_011312844	-	-	WP_011312675
**gamma-proteobacteria**										
Enterobacteriales	*Escherichia coli K-12 substr. MG1655*	complete	4,64	-	NP_415195	NP_417383	NP_417382	-	-	-
Acidithiobacillales	*Acidithiobacillus ferrooxidans ATCC 23270*	complete	2,98	-	-	ACK78396	ACK78157	-	-	ACK80750
Aeromonadales	*Aeromonas hydrophila ATCC 7966*	complete	4,74	-	YP_857731	YP_856252	YP_856253	-	-	-
Chromatiales	*Allochromatium vinosum DSM 180*	complete	3,53	-	-	WP_012972075	WP_012972076	-	-	WP_012969418
	Halorhodospira halophila SL1	complete	2,68	-	WP_011815011	WP_011813984	WP_011813985	-	-	-
Oceanospirillales	*Hahella chejuensis KCTC 2396*	complete	7,22	-	WP_011394734	WP_011395027	WP_011395028	-	-	WP_011399181
	Marinomonas mediterranea sp. nov. MMB-1T	complete	4,68	-	-	-	WP_013659887	-	-	WP_013662219
	Oceanospirillum sp. MED92	scaffold	3,87	-	EAR59540	EAR61114	EAR61113	-	EAR59978	-
Alteromonadales	*Psychromonas sp. CNPT3*	complete	3,05	-	WP_015465742	WP_015465961	WP_015465960	-	-	-
	Shewanella sp. ANA-3	complete	5,0	-	WP_011716127	WP_011718290	WP_011718289	-	-	-
	Alteromonas macleodii ATCC 27126	complete	4,65	-	WP_014948995	WP_014950376	WP_014950375	-	-	WP_014949273
Pasteurellales	*Actinobacillus pleuropneumoniae serovar 3 str. JL03*	complete	2,24	-	WP_005609181	WP_005619006	WP_012262784	-	-	-
Vibrionales	*Vibrio cholerae O395*	complete	3,02	ABQ21191	WP_012705998	WP_000132411	WP_000983782	-	-	-
Xanthomonadales	*Xanthomonas campestris pv. campestris str. ATCC 33913*	complete	5,08	-	-	NP_636189	NP_636190	-	NP_637995	NP_635869
	Stenotrophomonas maltophilia R551-3	complete	4,57	ACF52162	-	WP_012510105	WP_012510106	-	WP_004150679	WP_012512309
	Xylella fastidiosa M12	complete	2,48	-	-	WP_004084429	WP_004084431	-	-	WP_012337783
	Rhodanobacter fulvus Jip2	contig	3,88	EIL92977<br>EIL88490	-	EIL91924	EIL91923	-	EIL92972	WP_007081201
Legionellales	*Legionella pneumophila Corby*	complete	3,58	ABQ56323<br>ABQ54254	-	WP_011945283	WP_011945284	-	-	WP_011945219
Thiotrichales	*Francisella tularensis subsp. Holarctica LVS*	complete	1,90	-	-	WP_003015223	WP_003015224	-	-	WP_003015834
Pseudomonadales	*Moraxella catarrhalis RH4*	contig	1,84	-	-	EKF83221	EKF83694	-	EKF82976	-
	Psychrobacter lutiphocae DSM 21542	scaffold	3,18	-	-	WP_019673300	WP_019673301	-	WP_019672444	WP_019671918
	Pseudomonas aeruginosa PAO1	complete	6,26	NP_252879	-	NP_253910	NP_253908	-	-	NP_249346
Cardiobacteriales	*Dichelobacter nodosus VCS1703A*	complete	1,39	-	-	-	-	-	WP_012031324	-
	Cardiobacterium valvarum F0432	scaffold	2,55	-	-	-	-	-	WP_040389386	-
Salinisphaerales	*Salinisphaera shabanensis E1L3A*	complete	3,76	-	-	ERJ20799	ERJ20798	-	-	-
**EUKARYOTES**				**Coq6**						
Fungi	*Saccharomyces cerevisiae*			AAB61341	-	-	-	-	-	-
Metazoa	*Caenorhabditis elegans*			NP_505415.2	-	-	-	-	-	-
Amoebozoa	*Dictyostelium discoideum AX4*			XP_635209	-	-	-	-	-	-
Alveolata	*Paramecium tetraurelia strain d4-2*			XP_001347150	-	-	-	-	-	-
Euglenozoa	*Leishmania infantum JPCM5*			XP_001463206	-	-	-	-	-	-
Heterolobosea	*Naegleria gruberi strain NEG-M*			XP_002683632	-	-	-	-	-	-
Stramenopiles	*Phytophthora infestans T30-4*			XP_002898467	-	-	-	-	-	-
Viridiplantae	*Selaginella moellendorffii*			XP_002965486	-	-	-	-	-	-
	Chlorella variabilis			EFN59911	-	-	-	-	-	-
	Arabidopsis thaliana			AEE76872	-	-	-	-	-	-
	Physcomitrella patens			XP_001752808	-	-	-	-	-	-
