# Supplementary figures and images for: Evolution of Ubiquinone Biosynthesis: Multiple Proteobacterial Enzymes with Various Regioselectivities To Catalyze Three Contiguous Aromatic Hydroxylation Reactions
Source: mSystems. 2016 Aug 30;1(4):e00091-16. doi: 10.1128/mSystems.00091-16 (PMC5069965; doi:10.1128/mSystems.00091-16)

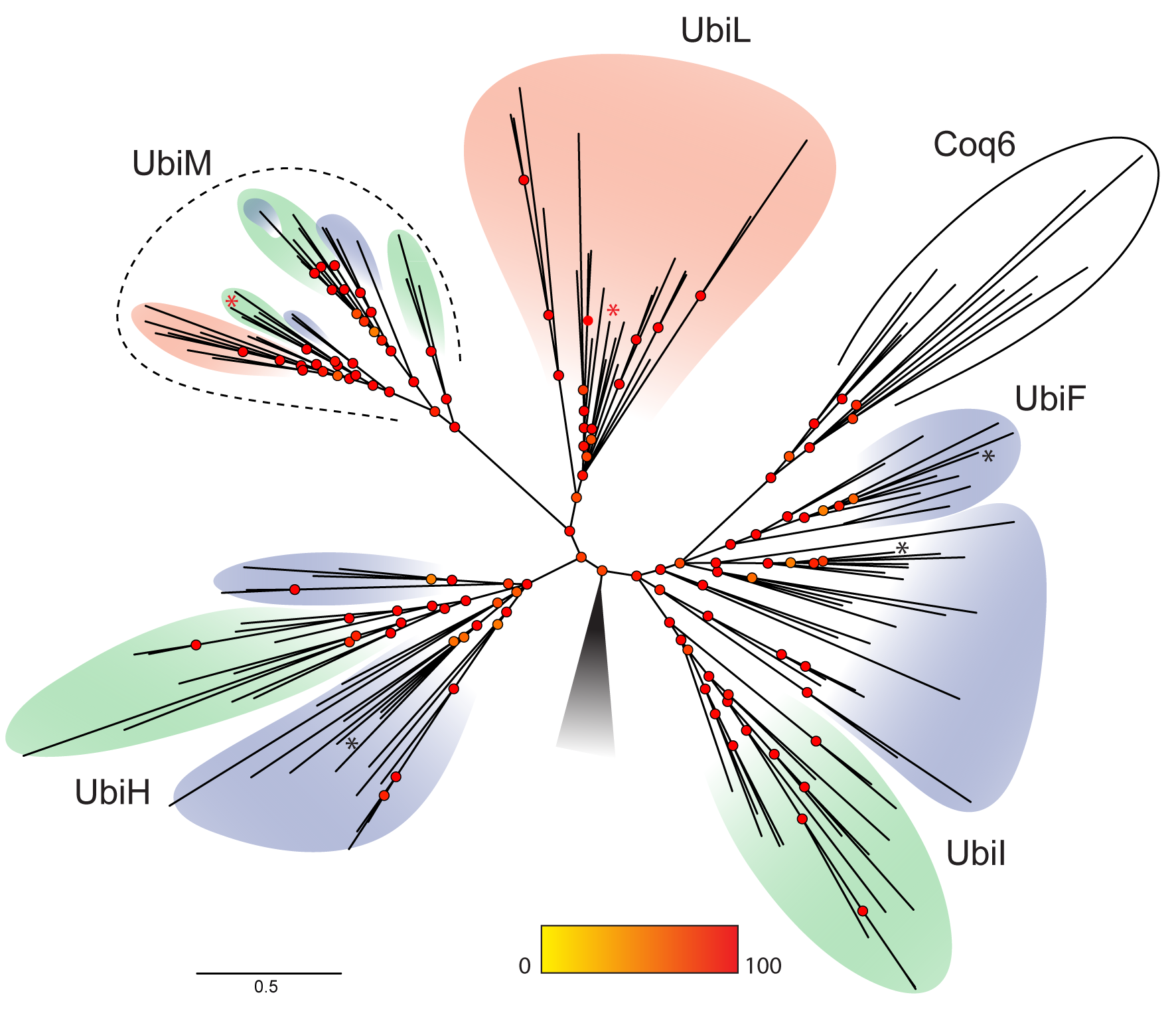

Supplement: Figure S1 [file sys004162048sf1.tif]

Figure S2

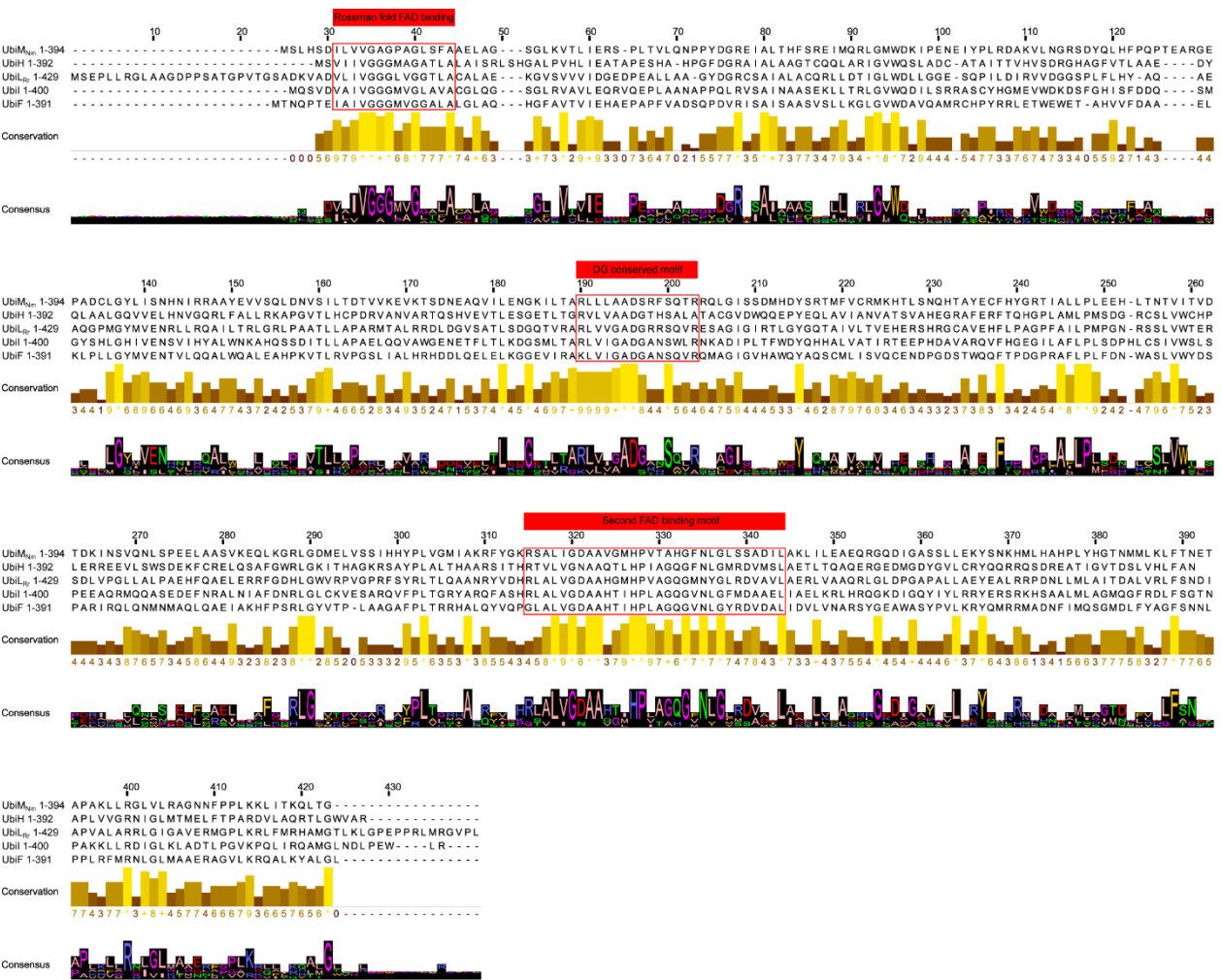

Supplement: Figure S2 [file sys004162048sf2.pdf]

**Figure S3**

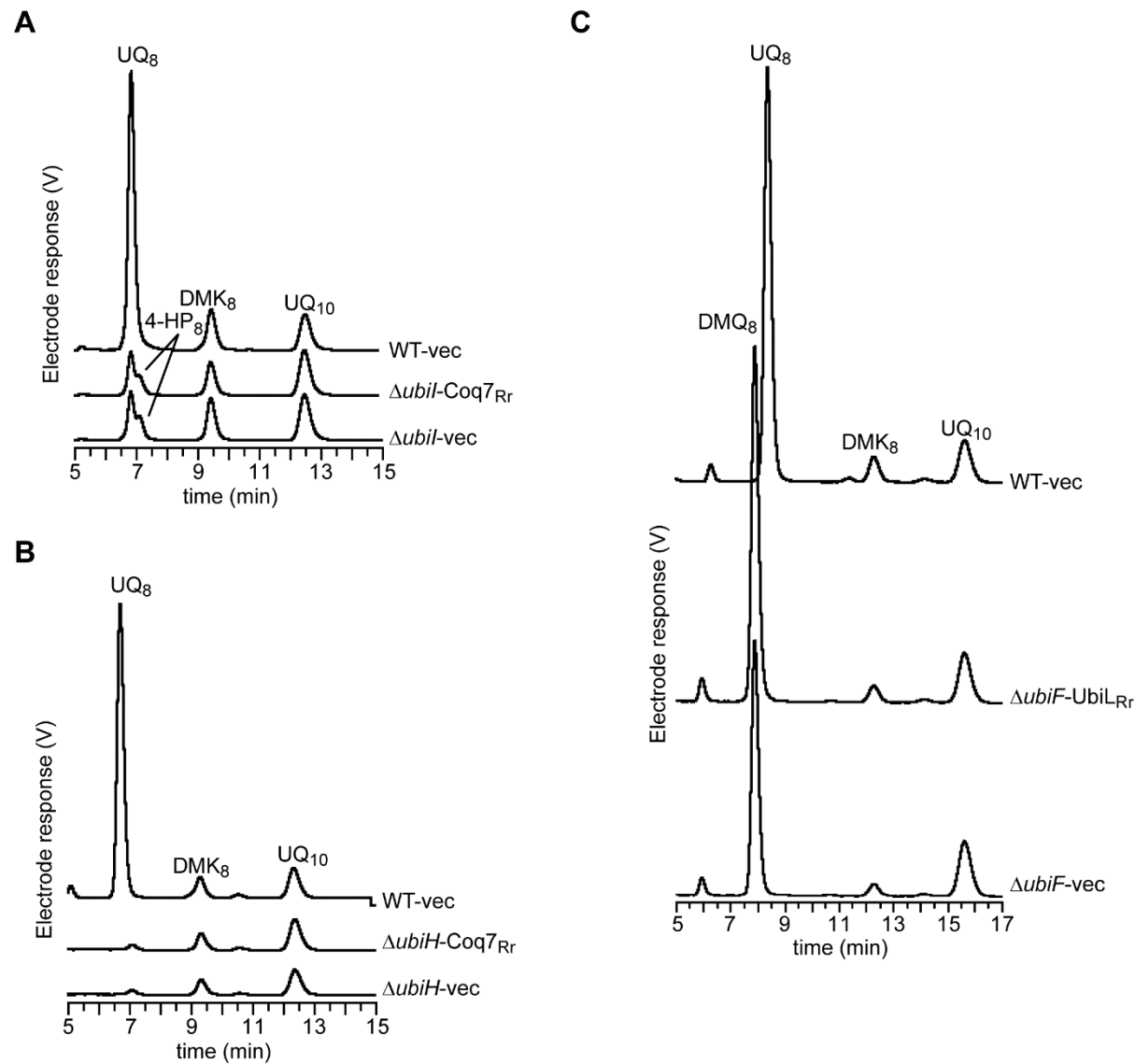

Supplement: Figure S3 [file sys004162048sf3.pdf]

**Figure S4**

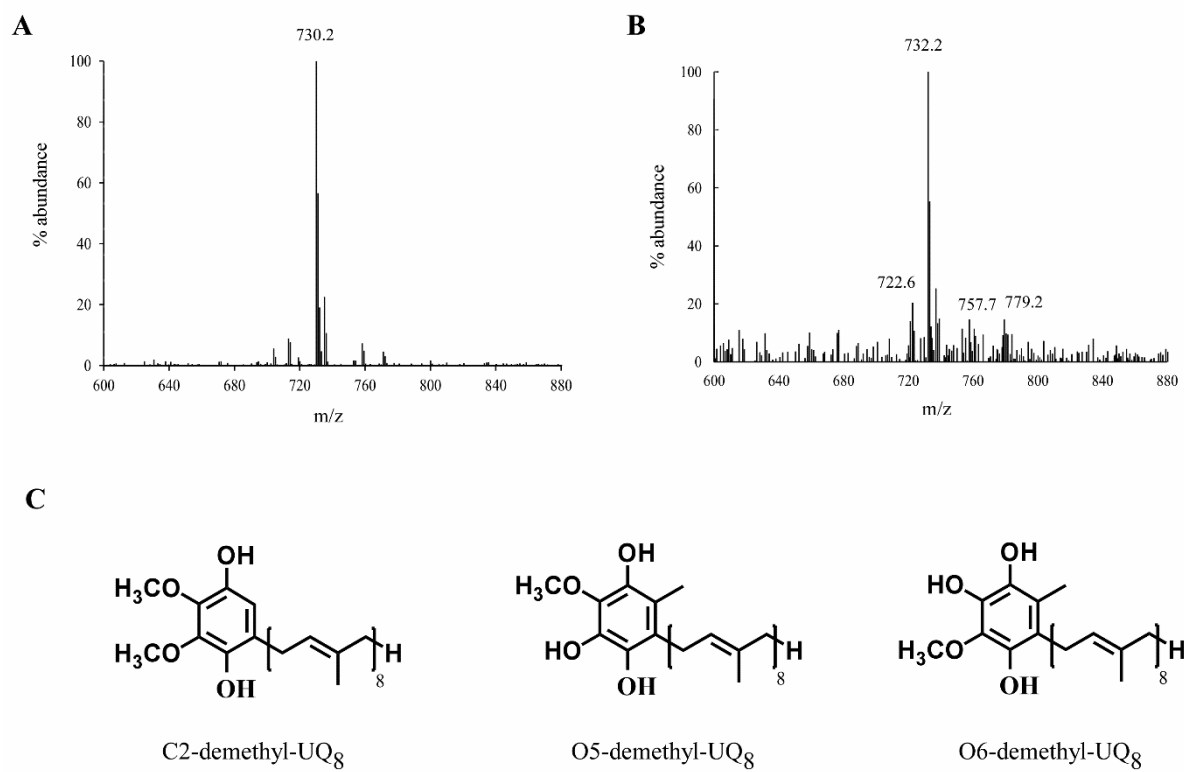

Supplement: Figure S4 [file sys004162048sf4.pdf]

Figure S5

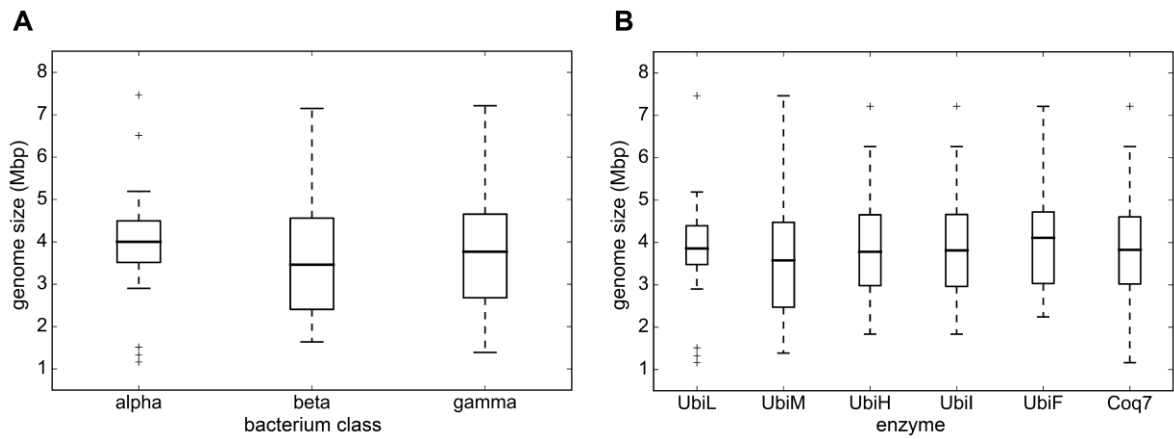

Supplement: Figure S5 [file sys004162048sf5.pdf]

Figure S6

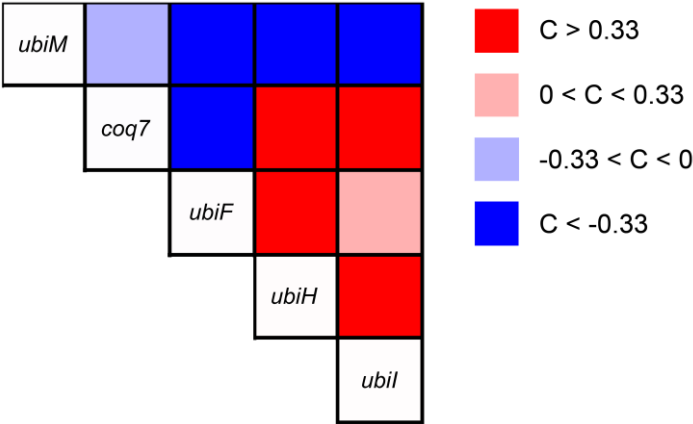

Supplement: Figure S6 [file sys004162048sf6.pdf]
