# Supplementary material for: Evolution of Ubiquinone Biosynthesis: Multiple Proteobacterial Enzymes with Various Regioselectivities To Catalyze Three Contiguous Aromatic Hydroxylation Reactions
Source: mSystems. 2016 Aug 30;1(4):e00091-16. doi: 10.1128/mSystems.00091-16 (PMC5069965; doi:10.1128/mSystems.00091-16)
Supplement: Table S2 [file sys004162048st8.pdf]

Table S2

	UbiF	UbiH	UbiI	UbiL	UbiM	Coq7
**Alpha-proteobacteria**						
Rhizobiales; Rickettsiales; Rhodospirilales; Kordiimonadales; Parvularculales; Rhodobacterales				+		+
Rhizobiales				++	+	
Rhodobacterales; Kiloniellales				++		
Rhodobacterales				+	+	
Rhodospirilales; Caulobacterales; Sphingomonadales				+	+	+
**Beta-proteobacteria**						
Neisseriales; Burkholderiales					+	
Burkholderiales			+		+	
Burkholderiales			+		+	+
Burkholderiales; Methylophylales		+	+		+	+
Burkholderiales; Rhodocyclales; Gallionellales; Nitrosomonadales; Hydrogenophylales		+	+			+
**Gamma-proteobacteria**						
Enterobacteriales; Chromatiales; Aeromonadales; Alteromonadales; Vibrionales; Pasteurellales	+	+	+			
Oceanospirillales; Alteromonadales	+	+	+			+
Oceanospirillales			+			+
Oceanospirillales	+	+	+		+	
Xanthomonadales; Pseudomonadales		+	+		+	+
Pseudomonadales		+	+		+	
Cardiobacteriales					+	
Salinisphaerales		+	+			
Pseudomonadales; Acidithiobacillales; Chromatiales; Xanthomonadales; Legionellales; Thiotrichales		+	+			+
