# Supplementary material for: Evolution of Ubiquinone Biosynthesis: Multiple Proteobacterial Enzymes with Various Regioselectivities To Catalyze Three Contiguous Aromatic Hydroxylation Reactions
Source: mSystems. 2016 Aug 30;1(4):e00091-16. doi: 10.1128/mSystems.00091-16 (PMC5069965; doi:10.1128/mSystems.00091-16)
Supplement: Table S3 [file sys004162048st9.pdf]

Table S3 Primers used in this study

Name	Sequence [a]	Purpose
5’wanner *ubiH*	aaatcgaagcgttgatggttgctgcgagaaagcaatgagcgCATATGAATATCCTCCTTA	Inactivation of *ubiHI*
3’wanner *ubiI*	agtgtgatgggtatcaaataaacaacagaggagaaatttGTGTAGGCTGGAGCTGCTTC	Inactivation of *ubiHI*
Fw-ubiLRr	TTTT<u>GAATTC</u>ATGAGCGAACCCCTGTTGCGCGGCC	Cloning *ubiL*$_{Rr}$
Rc-ubiLRr	TTTT<u>AAGCTT</u>TTACAAGGGAACCCCACGCATCAGGCGCGG	Cloning *ubiL*$_{Rr}$
Fw-coq7Rr	TTTT<u>GAATTC</u>ATGACCTCGCCGTCCTCGCGCACC	Cloning *coq7*$_{Rr}$
Rc-coq7Rr	TTTT<u>GGATCC</u>TCAGACCCGTTCGGAGACCCAGATCGCC	Cloning *coq7*$_{Rr}$
Fw-ubiMNm	TTTT<u>GAATTC</u>ATGAGTTTACACAGCGACATCCTCGTC	Cloning *ubiM*$_{Nm}$
Rc-ubiMNm	TTTT<u>GGATCC</u>TCAACCGGTCAGTTGTTTGGTAATCAG	Cloning *ubiM*$_{Nm}$

[a] Restriction sites are underlined.
