# Supplementary material for: Evolution of Ubiquinone Biosynthesis: Multiple Proteobacterial Enzymes with Various Regioselectivities To Catalyze Three Contiguous Aromatic Hydroxylation Reactions
Source: mSystems. 2016 Aug 30;1(4):e00091-16. doi: 10.1128/mSystems.00091-16 (PMC5069965; doi:10.1128/mSystems.00091-16)
Supplement: Table S4 [file sys004162048st10.pdf]

Table S4: *Escherichia coli* strains used in this study

**Strain**	**Relevant genotype**	**Construction**	**Source**
MG1655	Parental strain		Laboratory stock
Δ*ubiF*	Δ*ubiF::kan*	MG1655+P1/ JW0659, LB kan selection	(1)
Δ*ubiH*	Δ*ubiH::kan*	MG1655+P1/ JW2875, LB kan selection	(1)
Δ*ubiI*	Δ*ubiI::kan*	MG1655+P1/ JW2874, LB kan selection	(1)
Δ*ubiFc*	Δ*ubiF*	*ubiF* cured with pCP20, clone Kan^S	this work
Δ*ubiHc*	Δ*ubiH*	*ubiH* cured with pCP20, clone Kan^S	this work
Δ*ubiIc*	Δ*ubiI*	*ubiI* cured with pCP20, clone Kan^S	this work
Δ*ubiHI*	Δ*ubiHI::cat*	MG1655+P1/*ubiHI*, LB Cm selection	this work
Δ*ubiFHI*	Δ*ubiFc* Δ*ubiHI::cat*	*ubiF*c+P1/*ubiHI*, LB Cm selection	this work

1. **Hajj Chehade M, Loiseau L, Lombard M, Pecqueur L, Ismail A, Smadja M, Golinelli-Pimpaneau B, Mellot-Draznieks C, Hamelin O, Aussel L, Kieffer-Jaquinod S, Labessan N, Barras F, Fontecave M, Pierrel F.** 2013. ubiI, a New Gene in Escherichia coli Coenzyme Q Biosynthesis, Is Involved in Aerobic C5-hydroxylation. J Biol Chem **288:**20085-20092.
